# Background Parenchymal Enhancement on Contrast-Enhanced Mammography: Determinants, Technical Considerations, and Emerging Role as a Breast Cancer Risk Biomarker

**DOI:** 10.3390/cancers18152378

**Published:** 2026-07-23

**Authors:** Romuald Ferre, Cherie M. Kuzmiak

**Affiliations:** Division of Breast Imaging, Department of Radiology, University of North Carolina School of Medicine, 170 Manning Drive, Chapel Hill, NC 27599, USA

**Keywords:** contrast-enhanced mammography, background parenchymal enhancement, breast cancer risk, breast imaging, risk stratification

## Abstract

**Background/Objectives:** Background parenchymal enhancement (BPE) on contrast-enhanced mammography (CEM) represents enhancement of otherwise normal fibroglandular tissue. Although BPE is well established in breast MRI interpretation, its determinants, technical variability, diagnostic implications, and potential value as a breast cancer risk biomarker on CEM remain incompletely defined. This review summarizes the current evidence and outlines the steps required for responsible clinical translation. **Methods:** PubMed/MEDLINE was searched from database inception through April 2026 using terms related to CEM and BPE. Reference lists of eligible studies and relevant reviews were also screened. Human studies evaluating CEM-specific BPE in relation to biologic or hormonal determinants, breast density, technical factors, measurement methods, reproducibility, diagnostic performance, temporal variability, asymmetry, or breast cancer outcomes were synthesized narratively. **Results:** CEM BPE is influenced by age, menopausal status, menstrual and hormonal factors, lactation, endocrine therapy, breast density, contrast timing, view order, compression, positioning, imaging system, and post-processing. Because CEM is a projection-based technique acquired over several minutes, its BPE should not be considered physiologically interchangeable with MRI BPE. Moderate or marked BPE may reduce lesion conspicuity or mimic abnormal enhancement, particularly when asymmetric. Early studies suggest that higher CEM BPE may be associated with prevalent or subsequent breast cancer after adjustment for established risk factors, but findings remain heterogeneous and derive predominantly from retrospective, single-center cohorts. Quantitative and artificial-intelligence approaches are promising but require technical normalization, reproducibility testing, multicenter validation, and linkage to clinically meaningful outcomes. **Conclusions:** CEM BPE is a functional imaging feature shaped by patient biology, tissue composition, acquisition technique, and reader assessment. It should currently be reported and interpreted in context but should not independently alter screening, biopsy, or risk-management decisions. Translation into a clinically useful biomarker will require standardized measurement, longitudinal prognostic validation, demonstration of added value beyond established risk models, and prospective evidence that BPE-informed care improves outcomes without producing excessive harms or costs.

## Simple Summary

This review explains that background parenchymal enhancement (BPE) on contrast-enhanced mammography (CEM) is not just “background noise.” It reflects a mix of patient biology, breast density, hormones, technical acquisition factors, and reader interpretation. Higher BPE may be linked to increased breast cancer risk, but the evidence is still early and inconsistent. Therefore, BPE should be reported and interpreted in context, but it should not yet change screening, biopsy, or risk-management decisions by itself. Future studies need standardized measurement, AI/quantitative tools, and long-term validation.

## 1. Introduction

Contrast-enhanced mammography (CEM) is no longer used only as a problem-solving test. It is increasingly applied in diagnostic assessment, preoperative staging, and supplemental screening. With a dedicated CEM lexicon, updated practice guidance, and new dense-breast screening data, contrast-enhanced breast imaging is now part of the broader conversation about how to detect and risk-stratify breast cancer beyond MRI [1,2,3,4,5]. In that context, background parenchymal enhancement (BPE) deserves closer attention. What was once easy to dismiss as background ‘noise’ may affect interpretation and may also reflect underlying breast biology. Although earlier studies commonly used the term contrast-enhanced spectral mammography (CESM), CEM is used consistently throughout this review.

The expanding use of CEM is clinically important because it brings functional contrast information into settings where conventional mammography has historically provided primarily structural information. This shift creates new opportunities, but it also requires radiologists to understand which enhancing findings represent lesions and which represent normal parenchymal physiology. In routine practice, BPE is encountered before a lesion is characterized: it forms the visual background against which enhancement is detected, judged, and compared across views. A review of CEM BPE is therefore timely not only for readers who already use CEM, but also for practices considering CEM as an alternative or complement to MRI in selected diagnostic or screening pathways [1,2,5].

On CEM, BPE is the enhancement of normal fibroglandular tissue seen on recombined or subtracted images. In practice, it is usually described with the same qualitative categories used for MRI—minimal, mild, moderate, and marked [1,3]. Because CEM depends on iodinated contrast uptake, BPE can mask subtle enhancement, resemble nonmass enhancement, or produce asymmetry that leads to additional workup. At the same time, experience from breast MRI suggests that background enhancement can be a marker of hormonally influenced vascular and epithelial activity, rather than only a source of interpretive difficulty [6,7,8,9,10].

For the purposes of clinical interpretation, the most useful approach is to describe BPE as a property of the examination rather than as a diagnosis. This distinction helps avoid two common errors. The first is dismissing all background enhancement as irrelevant technical noise. The second is treating a visually high-BPE category as proof of abnormal tissue biology in an individual patient. A balanced interpretation recognizes that BPE can simultaneously reduce lesion conspicuity, reflect hormonal or vascular influences, and remain insufficiently validated as an independent management trigger. This review therefore emphasizes determinants, practical interpretation, and research gaps, with particular attention to the difference between promising biomarker evidence and evidence strong enough to change patient care.

## 2. Review Methodology

This article was designed as a structured narrative review. PubMed/MEDLINE was searched from database inception through April 2026 using terms related to contrast-enhanced mammography, including “contrast-enhanced mammography,” “contrast-enhanced spectral mammography,” “contrast-enhanced digital mammography,” “CEM,” “CESM,” and “CEDM,” combined with terms related to background parenchymal enhancement, including “background parenchymal enhancement,” “parenchymal enhancement,” and “BPE.” Reference lists of eligible studies and relevant reviews were also screened to identify additional publications. The supplied PubMed search export contained 2656 records and included many articles unrelated to CEM-specific BPE, such as studies focused on lesion enhancement, breast MRI, contrast-enhanced CT or ultrasound, technical performance, radiation dose, artifacts, biopsy, staging, and screening outcomes. After targeted assessment, 35 publications directly evaluating BPE on CEM were included in the principal narrative synthesis. These studies addressed CEM–MRI agreement, hormonal and patient-related determinants, breast density and menopausal status, menstrual-cycle effects, vendor and acquisition-related variability, visual and automated assessment, interreader reproducibility, temporal fluctuations, asymmetric BPE, diagnostic performance, and associations with breast cancer risk. This is further detailed in Supplementary Materials (Figure S1 and Table S1).

Eligible publications were human studies reporting CEM-specific BPE data related to biologic or clinical determinants, technical influences, visual or quantitative assessment, reproducibility, diagnostic impact, longitudinal variability, asymmetry, or breast cancer risk. MRI studies were included selectively when they provided relevant biologic or methodological context, but MRI-only evidence was not considered direct validation of CEM BPE. Case reports, conference abstracts without full-text publication, non-breast studies, animal and phantom studies, purely technical or dosimetric investigations without patient-level BPE data, and articles addressing lesion enhancement without evaluation of normal background parenchymal enhancement were excluded RF screened all records, and CMK reviewed all included and uncertain records. Differences in eligibility assessment were resolved by consensus. Because of substantial heterogeneity in study populations, clinical indications, CEM acquisition protocols, BPE assessment methods, and reported endpoints, quantitative meta-analysis was not considered appropriate. Findings were therefore synthesized narratively according to the principal biologic, technical, diagnostic, reproducibility, and risk-related themes.

## 3. Conceptualizing BPE on CEM

Three points are useful when approaching BPE on CEM. First, the finding is neither purely biologic nor purely technical. From a biologic standpoint, BPE reflects contrast distribution in fibroglandular tissue and is influenced by perfusion, vascular permeability, hormonal state, and the amount of tissue available to enhance. From a technical standpoint, the amount of BPE a reader sees depends on iodine delivery, time from injection to image acquisition, compression, image processing, view order, and the projection-based nature of mammography.

A useful conceptual model is to separate the source of enhancement from the way enhancement is displayed. The source is the patient’s normal fibroglandular tissue and its vascular response after iodinated contrast administration. The display is the final recombined CEM image, which is shaped by acquisition timing, compression, post-processing, and the two-dimensional summation of breast tissue. These layers are difficult to separate visually, which explains why the same patient may appear to have different degrees of BPE across examinations or even across views. Recognizing this layered origin of BPE is especially important when interpreting borderline cases, where a category such as mild versus moderate may depend as much on technique and timing as on underlying tissue biology [11,12,13,14,15,16,17,18,19,20].

Second, CEM BPE should not be treated as the same entity as MRI BPE. MRI provides volumetric, dynamic, multiparametric data. CEM provides dual-energy projection images acquired during a relatively narrow post-contrast window. MRI can show three-dimensional enhancement distribution and kinetic behavior, whereas CEM collapses tissue along a projection plane and usually does not offer true kinetic characterization [3,11,12]. Similar qualitative labels are useful for reporting, but they should not imply a one-to-one physiologic equivalence between the two modalities.

Third, BPE is related to, but distinct from, mammographic density. Density describes the proportion of fibroglandular tissue visible on low-energy or conventional mammographic images. BPE describes how that tissue enhances after contrast administration. The two often overlap because dense breasts contain more fibroglandular substrate, but they are not interchangeable. Several CEM studies have found associations between density and BPE; early risk-focused analyses suggest that BPE may add information beyond density, although this remains unproven in routine care [12,13,14,15,16,17,18].

This distinction also affects how BPE should be discussed with clinicians and patients. Density is already incorporated into screening discussions because it influences both cancer risk and mammographic sensitivity. BPE is different: it may add information about active enhancement in the tissue that is present, but it is not yet incorporated into validated clinical risk models for CEM.

**Figure 1:**
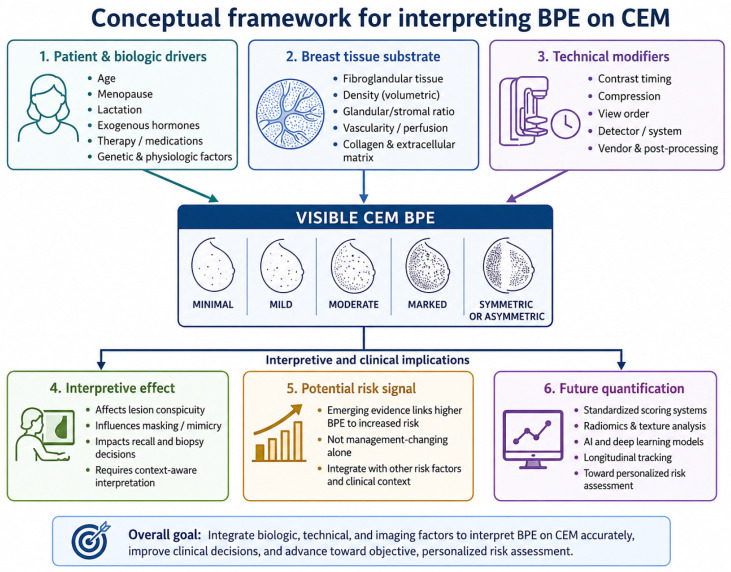
Conceptual framework for interpreting BPE on CEM. Visible BPE reflects the interaction of patient biology, breast tissue substrate, acquisition technique, and reader assessment. It can influence interpretation and may carry a risk signal, but current evidence is not sufficient to support management changes based on BPE alone.

This distinction has practical consequences. In patients with dense breasts, minimal and marked BPE represent different imaging contexts because they may affect lesion conspicuity and diagnostic confidence differently. However, neither pattern currently supports a CEM-specific change in screening, biopsy, or risk-management decisions by itself. Thus, the distinction is presently relevant to image interpretation rather than patient management. As summarized in Figure 1, visible CEM BPE reflects the interaction of patient biology, fibroglandular tissue substrate, acquisition technique, and reader assessment. In the following sections, we review the biologic, clinical, and technical determinants of CEM BPE, summarize evidence regarding its effects on interpretation and its association with breast cancer risk, and propose a practical framework for its reporting and clinical interpretation while identifying the validation steps required for future biomarker use.

The value of BPE will depend on whether it improves prediction beyond factors that are already known and actionable [12,13,14,15,16,17,18,19,20,21,22,23].

## 4. Biologic and Clinical Determinants

### 4.1. Age, Menopausal Status, and Hormonal Exposure

Across the literature, the most consistent biologic signal is hormonal. Breast MRI studies have shown that BPE varies with menstrual phase, menopausal status, exogenous hormone exposure, and endocrine therapy [24,25,26,27,28,29,30,31]. CEM studies are now showing the same general pattern. Higher BPE has been linked with younger age, premenopausal status, and hormonally active states, and more recent work has reported lower BPE after menopause and tamoxifen therapy and higher BPE during lactation and hormone replacement therapy [13,15,16,18,19].

Hormonal interpretation should also account for timing and treatment history. In premenopausal patients, breast vascularity and epithelial activity can vary across the menstrual cycle, so a single CEM examination may capture only one point within a dynamic physiologic range. In patients using exogenous hormones, in lactating patients, and in patients receiving endocrine therapy, BPE may reflect a changing hormonal environment rather than a stable baseline characteristic. These considerations are not reasons to avoid reporting BPE. Instead, they support contextual language and careful comparison with prior studies when available. They also explain why research cohorts should record hormonal exposures in detail, because unmeasured hormonal factors can confound apparent associations between BPE and cancer outcomes [6,13,15,16,18,19,28,29,30,31].

These observations are clinically important because they argue against treating BPE as a fixed anatomic trait. Marked BPE in a lactating patient, a patient taking hormone therapy, or an actively cycling premenopausal patient may not carry the same implication as persistent marked BPE in a postmenopausal patient. Reporting should therefore avoid implying that BPE is abnormal by default. It is better framed as a background imaging feature whose significance depends on the clinical setting.

### 4.2. Breast Density and Fibroglandular Tissue

Breast density is one of the most reproducible correlates of CEM BPE. Sogani et al. found that BPE on CEM and MRI was most often minimal or mild, but it was associated with density and hormonal factors [11]. Subsequent CEM studies have also reported higher BPE in denser breasts, although the strength of the association has varied by population and method [13,14,15,16,17,18]. This relationship is intuitive: more fibroglandular tissue creates more substrate that can enhance.

Still, density and BPE answer different questions. Density is primarily structural and has implications for masking. BPE is functional and reflects contrast uptake, vascularity, and hormonal activity. This distinction helps explain why BPE may remain informative after density adjustment in early risk studies, and why a dense breast does not always show high BPE.

Another practical implication is that density may amplify the visibility of BPE without fully explaining it. Dense tissue provides a larger volume of potential enhancing substrate, yet enhancement intensity and distribution can differ markedly among patients with similar density categories. This variability may reflect differences in vascularity, permeability, stromal composition, hormone responsiveness, prior therapy, and image acquisition. For that reason, studies that adjust for density are particularly important. They ask whether BPE is merely a visual consequence of having more fibroglandular tissue or whether it captures additional functional information. Early CEM risk studies suggest the latter possibility, but the evidence remains preliminary and should be interpreted cautiously [14,15,16,17,18,22,23].

### 4.3. Temporal Variability

Recent longitudinal work is also a reminder that CEM BPE can change from one examination to the next. In one analysis, reported BPE varied more across serial CEM examinations than mammographic density did [20]. That variability creates a challenge for risk prediction based on a single examination. It also creates an opportunity: if change in BPE can be measured reliably, it may someday help characterize hormonal exposure, preventive therapy response, or shifts in tissue biology.

Temporal variability has two sides. On one hand, it limits the reliability of one-time BPE classification as a durable marker of long-term risk. A category assigned on a single examination may be affected by menstrual timing, medication changes, lactation, scanner protocol, compression, or reader variability. On the other hand, change itself may eventually become informative. A sustained decrease in BPE after endocrine therapy, for example, could be explored as a marker of reduced hormonal stimulation, whereas persistent or increasing BPE might identify a subgroup requiring closer study. Such uses remain investigational, but they highlight why serial CEM datasets should preserve acquisition details and patient-level hormonal information rather than reducing BPE to a single static label [19,20].

Until reproducibility thresholds are better defined, radiologists should be cautious about attaching long-term risk meaning to one BPE category. Persistent marked BPE, or clearly asymmetric BPE that persists over time, may deserve careful attention. Management decisions, however, should still rest on lesion morphology, clinical risk, comparison examinations, and established screening guidance.

## 5. Technical Determinants and Interpretive Pitfalls

CEM also has modality-specific technical features that can change how much BPE is visible. Unlike MRI, which can acquire dynamic bilateral images, CEM obtains compressed projection images in sequence. As a result, the apparent enhancement of normal parenchyma can vary by breast, view, and time after contrast injection. View order, acquisition timing, compression force, and vendor-specific recombination algorithms can all influence the final appearance of BPE [12,16,17,32,33].

Projection imaging also means that normal structures overlap. Enhancing parenchyma, vessels, scars, and postoperative or post-radiation changes can be superimposed, making the apparent extent of BPE dependent on positioning and view. Unlike MRI, CEM usually cannot resolve enhancement into a volumetric distribution or confirm whether a subtle area represents linear, segmental, regional, or diffuse enhancement in three dimensions. This limitation is central to the interpretation of nonmass-like background patterns. When the recombined image shows patchy or regional enhancement, readers must integrate the low-energy image, the contralateral breast, prior examinations, and any sonographic correlate before assigning pathologic significance [3,11,12,32,33].

Compression deserves particular attention. By altering vascular volume and tissue thickness, compression may affect both lesion conspicuity and background enhancement. Ferrara et al. reported only fair-to-moderate agreement between CEM and MRI BPE assessment and identified compression force and mammographic density as CEM-specific influences [12]. In practical terms, this means CEM BPE is not simply MRI BPE translated into another modality.

Timing after contrast injection is another common source of variability. CEM acquisition usually extends over several minutes, during which BPE may increase. Differences in timing can create apparent asymmetry or change the conspicuity of subtle enhancing findings. Before recommending extensive workup for apparent asymmetric BPE, readers should consider timing, positioning, compression, treatment history, and whether the pattern represents true tissue asymmetry.

Artifacts can further complicate interpretation. Motion, misregistration, skin enhancement, vessels, post-surgical change, post-radiation change, and subtraction artifacts may mimic BPE or make it appear more extensive than it is [32,33]. For this reason, any discussion of CEM BPE as a possible biomarker should be balanced by an equally practical discussion of its day-to-day interpretive pitfalls.

A practical reading strategy begins with global assessment before focal analysis. Readers can first decide whether BPE is symmetric or asymmetric, whether it is diffuse or regional, and whether the degree of enhancement is similar across views obtained at different times after injection. They can then inspect the low-energy images for architectural distortion, calcifications, masses, or postsurgical changes that might explain focal enhancement. Finally, any suspicious area should be judged by lesion morphology rather than by the BPE category alone. This stepwise approach reduces the chance of overcalling physiologic enhancement while still protecting against the opposite error: allowing marked background enhancement to obscure a clinically meaningful lesion [3,25,32,33].

## 6. Evidence Linking CEM BPE to Breast Cancer Outcomes

### 6.1. Association with Cancer Present at the CEM Examination

Evidence directly linking CEM BPE with breast cancer risk is still limited, but it is worth taking seriously. In a cohort of 516 women undergoing CEM, Sorin et al. found that moderate-to-marked BPE was associated with increased odds of breast cancer after adjustment for age, family history, and mammographic density. The association was stronger in the screening subgroup, raising the possibility that BPE may capture risk-related information not fully reflected by density [22].

The biologic rationale for a risk association is plausible. BPE may reflect vascular permeability, hormone-responsive epithelium, stromal activity, and inflammatory or angiogenic signaling within otherwise normal-appearing tissue. Similar concepts have been explored more extensively with MRI, where higher BPE has been associated with future breast cancer risk in several settings. CEM cannot be assumed to reproduce MRI risk information, because the modalities measure enhancement differently, but MRI experience provides a framework for why background enhancement may be more than a nuisance finding. The key question for CEM is whether its simpler, projection-based acquisition can capture a sufficiently reproducible signal to improve risk stratification in real-world screening and diagnostic populations [6,7,8,9,10,22,23].

### 6.2. Prediction of Subsequent Breast Cancer

Xu et al. examined the question from a longitudinal perspective in high-risk women after baseline CEM. Increasing BPE category was associated with a dose–response increase in subsequent breast cancer, and women with more than minimal BPE had substantially higher odds of developing cancer during follow-up [23]. This design is especially informative because it addresses future cancer development, not simply the presence of cancer at the time of a diagnostic examination.

The literature is not uniform. Yu et al. did not find a significant relationship between BPE category and malignancy in a diagnostic CEM cohort [24]. That result should not be viewed as a direct refutation of the risk studies. Diagnostic cohorts often answer a different question: whether a current finding is malignant in a population already referred for symptoms or workup. Risk studies ask whether enhancement in otherwise normal parenchyma predicts future cancer. These questions overlap, but they are not the same.

### 6.3. Influence of BPE on Diagnostic Performance and Masking

For clinical readers, this distinction is critical. A biomarker candidate can be worthy of study, standardized reporting, and quantitative development without being ready to guide an individual patient’s screening pathway. At present, moderate or marked CEM BPE should not automatically lead to MRI referral, shortened screening interval, biopsy, or genetic testing. Those decisions should continue to depend on established clinical risk factors, breast density, genetic and family history, lesion findings, and patient preferences. The most appropriate current use of BPE is therefore interpretive and contextual: it helps explain examination limitations and may contribute to future models, but it should not be used as an isolated marker of cancer risk in daily practice. Taken together, the data support CEM BPE as a promising risk biomarker candidate, not as a stand-alone clinical decision tool. The early findings justify consistent reporting and further study. They do not yet support changing screening intervals, recommending MRI, or altering biopsy thresholds on the basis of BPE alone.

Representative CEM studies addressing biologic and technical determinants, cross-modality agreement, temporal variability, diagnostic performance, and breast cancer risk are summarized in Table 1.

## 7. BPE as an Interpretive Challenge

Marked BPE can make CEM interpretation less confident. It may obscure subtle enhancing lesions, including low-conspicuity tumors, nonmass enhancement, ductal carcinoma in situ, and invasive lobular carcinoma. Conversely, patchy or asymmetric BPE can resemble disease and lead to recall, targeted ultrasound, short-interval follow-up, MRI correlation, or biopsy. The practical challenge is not whether BPE matters, but how to respond to it without overcalling background enhancement.

The risk of overcalling is especially relevant in supplemental screening, where the goal is to detect mammographically occult cancer without creating excessive false-positive workup. Patchy BPE may generate recalls, targeted ultrasound examinations, short-interval follow-up recommendations, or biopsies that ultimately prove benign. Conversely, if the reader becomes accustomed to dismissing enhancement in a high-BPE breast, subtle disease may be overlooked. The best safeguard is disciplined pattern recognition: true lesions should demonstrate a definable morphology, reproducible location, or convincing correlate, whereas background enhancement usually lacks a discrete mass effect and may vary with view, timing, or comparison examination. This distinction is sometimes difficult but explicitly applying it can improve consistency across readers and practices [21,25].

**Figure 2:**
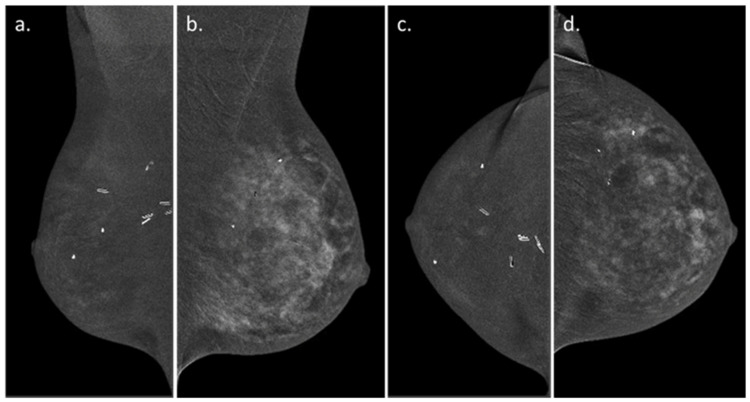
Asymmetric background parenchymal enhancement following unilateral breast irradiation. Recombined mediolateral oblique (**a**,**b**) and craniocaudal (**c**,**d**) CEM images demonstrate marked left-sided and minimal right-sided BPE in a patient with previous right breast-conserving treatment and irradiation. This example illustrates a treatment-related asymmetric background pattern rather than a validated risk or management threshold. Reproduced/adapted from Reference [21].

Asymmetric BPE deserves a separate mention. Recent institutional experience suggests that asymmetric BPE can prompt additional workup and may occur in several settings, including prior treatment and benign physiologic asymmetry [21]. The central interpretive question is whether the enhancement behaves like a lesion, whether it has a correlate on low-energy mammography or ultrasound, and whether it persists on prior or subsequent examinations (Figure 2).

When BPE is high, readers should also perform a technical check before assigning too much significance to the finding. Contrast timing, compression, motion, subtraction quality, vascular overlap, surgical or radiation change, and symmetry across views should all be considered. This approach is safer than allowing the BPE category alone to drive interpretation. A practical framework for interpreting common CEM BPE patterns within current clinical practice is provided in Table 2.

## 8. Reporting and Current Clinical Integration

### 8.1. Structured Reporting and Clinical Communication

At present, the most appropriate role for BPE on CEM is as a structured descriptive imaging feature, rather than as an independent risk marker or management determinant. Consistent documentation may improve communication between radiologists and referring clinicians, support comparison across examinations, facilitate quality assurance, and create research-ready data. However, the reported BPE category should not, by itself, change an individual patient’s screening, diagnostic, or biopsy pathway [1,3].

A structured report could document four elements:

- Background parenchymal enhancement: minimal, mild, moderate, or marked; symmetric or asymmetric.
- Interpretive impact: no meaningful limitation or possible limitation in the assessment of subtle enhancement, particularly nonmass enhancement.
- Relevant context: acquisition timing, positioning, compression, hormonal status, lactation, endocrine therapy, or prior surgery or radiation, when known and relevant to interpretation.
- Management statement: BPE alone does not alter management; any additional evaluation is based on focality, persistence, morphology, or a corresponding finding on low-energy mammography, ultrasound, or another imaging examination.

Relevant clinical or technical context should be included only when it is known and reasonably likely to affect interpretation. The report should avoid speculative attribution of BPE to hormonal or treatment-related factors when this information is unavailable.

A possible report statement is:

*“Moderate symmetric background parenchymal enhancement is present and may mildly reduce sensitivity for subtle nonmass enhancement. No suspicious enhancing lesion is identified. No change in management is recommended on the basis of BPE alone.”*

When enhancement appears asymmetric, the radiologist should distinguish a background pattern from a discrete enhancing abnormality. Apparent asymmetry should first be assessed for differences in acquisition timing, compression, positioning, breast thickness, vascular overlap, and prior surgery or radiation. Comparison with the contralateral breast, low-energy images, prior CEM examinations, and targeted ultrasound may help determine whether the finding represents background enhancement or a focal lesion.

Additional evaluation should not be recommended solely because BPE is asymmetric or categorized as moderate or marked. Rather, further assessment should be driven by persistent focal enhancement, suspicious morphology or distribution, interval change, or a convincing mammographic or sonographic correlate. When additional imaging is recommended, the report should state the specific imaging feature prompting the recommendation rather than attributing the decision to the BPE category alone.

Because patients may read reports containing unfamiliar terminology, a brief explanatory sentence may be helpful, such as: “Background parenchymal enhancement reflects contrast uptake in normal breast tissue.” When additional evaluation is recommended, the report should link the recommendation to a focal or asymmetric imaging feature rather than to the BPE category alone.

### 8.2. Radiologist Training and Quality Assurance

Training should address both accurate categorization and the prevention of interpretive errors. A CEM BPE teaching set should include the full range of BPE appearances, examinations acquired at different post-contrast times, compression- and positioning-related differences, vascular overlap, motion and subtraction artifacts, and cases involving lactation, exogenous hormone use, endocrine therapy, prior surgery, and radiation treatment.

Readers should be trained to distinguish diffuse, patchy, or bilateral background enhancement from a reproducible focal abnormality. Interpretation should incorporate the low-energy images, both standard views, the contralateral breast, prior examinations, and ultrasound findings when available. Particular attention should be given to asymmetric patterns and to examinations in which post-treatment change or technical artifacts may mimic enhancement.

Practices performing CEM could incorporate periodic reader-calibration exercises and interreader audits. These activities may be especially useful for category boundaries, such as mild versus moderate BPE, and for distinguishing asymmetric background enhancement from focal nonmass enhancement. The purpose of calibration is not to establish a new clinical threshold, but to improve consistency in interpretation, reporting, communication, and data collection.

Quality-assurance programs could also monitor the frequency of unclassifiable examinations, disagreement between readers, recalls attributed to possible asymmetric BPE, and the effect of technical factors such as acquisition timing or motion. Cases with substantial disagreement or unexpected outcomes could be reviewed in multidisciplinary or peer-learning sessions.

### 8.3. Exploratory Integration with Existing Risk Models

BPE should initially be investigated as an additional candidate variable within established breast cancer risk frameworks rather than as a replacement for them. Studies should determine whether visual or quantitative CEM BPE provides information beyond age, breast density, family history, genetic susceptibility, reproductive and hormonal factors, prior breast disease, and existing model-based risk estimates [22,23].

Model development and evaluation should be performed in separate populations, followed by external validation in independent cohorts. Evaluation should extend beyond discrimination alone and include calibration, reproducibility, clinically relevant reclassification, and the consequences of moving patients across established risk thresholds. Analyses should also consider whether any apparent association between BPE and risk is independent of breast density, menopausal status, hormone exposure, treatment, and technical acquisition factors.

An appropriate early approach would be a silent-mode study, in which BPE-informed risk estimates are calculated but are not displayed to clinicians and do not alter patient care. This would allow investigators to assess the incremental contribution, stability, calibration, and potential unintended consequences of adding BPE before patient-facing implementation.

Until such evidence is available, CEM BPE should remain a structured reporting and research variable. It should not independently prompt MRI referral, shortened screening intervals, preventive medication, genetic testing, biopsy, or other changes in management.

## 9. Future Directions

### 9.1. From Association to a Clinically Reliable Biomarker

Translation of CEM BPE from a promising association to a clinically reliable biomarker requires sequential validation rather than a single multicenter study [34]. Before clinical use is considered, the intended role of BPE must first be defined. BPE could function as a general marker of future breast cancer risk, a marker of masking and reduced examination sensitivity, a local marker of tissue susceptibility, or a longitudinal marker of hormonal or preventive-treatment response. These potential uses require different populations, endpoints, and thresholds. A phased validation pathway can help distinguish exploratory association from evidence sufficient to support clinical action (Table 3).

### 9.2. Objective Quantitative Measurement and AI

An objective CEM BPE tool should do more than reproduce the four visual BI-RADS categories. Training an algorithm only to imitate subjective reader labels could encode existing interreader variability rather than solve it. The preferred objective is a continuous, technically normalized measurement linked to reproducibility and clinically meaningful outcomes.

Existing CEM-AI studies demonstrate the feasibility of automated image analysis but also highlight the gap between lesion analysis and objective BPE measurement. Jailin et al. developed a deep-learning model using 1673 patients and found that combining low-energy and recombined images improved cancer detection and breast classification, achieving an overall breast-level AUC of 0.964. Importantly, performance decreased in examinations with higher BPE: the classification AUC was 0.986 in the low-BPE group and 0.919 in the high-BPE group, while the false-positive rate at 90% sensitivity increased from 0.080 to 0.416 per image. However, BPE was used as a visually assigned subgroup rather than measured as a continuous quantitative signal, indicating that current algorithms may be affected by BPE without directly quantifying it [35].

Related commentaries have described similarly promising results for automated lesion detection, segmentation, and classification on CEM. Bahl and Do highlighted models achieving classification AUCs of approximately 0.93–0.95 but noted persistent limitations in the detection of calcifications, testing on normal examinations, and comparison with radiologists [36]. Zhang and Mann reviewed models integrating bilateral, dual-view, and dual-energy information, with internal and external AUCs of approximately 0.96 and 0.92, respectively, while emphasizing the need for multicenter training and improved performance for BI-RADS 4 lesions and microcalcifications [37]. These studies support the potential of AI for CEM interpretation, but they primarily address lesion detection and classification rather than technically normalized BPE measurement.

A quantitative BPE pipeline would ideally include several steps. First, the system should import both image data and relevant DICOM or acquisition metadata, including contrast dose and concentration, injection rate, time from injection to each view, acquisition order, breast thickness, compression force, vendor platform, and recombination software. Second, it should segment the breast and fibroglandular tissue on low-energy and recombined images. Third, it should identify or exclude vessels, skin enhancement, motion and subtraction artifacts, known lesions, surgical beds, and irradiated tissue when these would bias measurement. Fourth, the measured enhancement should be normalized for technical factors such as post-contrast timing, breast thickness, compression, exposure, and vendor-specific processing.

Candidate outputs include mean or percentile enhancement intensity, enhancing fibroglandular tissue fraction, total enhancing parenchymal area or volume surrogate, right–left asymmetry, regional distribution, textural or spatial heterogeneity, consistency across views, and longitudinal change between examinations. Measurements should be accompanied by an uncertainty or quality score, particularly when motion, incomplete positioning, strong vascular overlap, or post-treatment changes reduce reliability.

Validation should include repeatability within the same examination, test–retest reliability across examinations, comparison with expert consensus, and external evaluation across institutions and vendors. Algorithms should also be tested separately in premenopausal and postmenopausal patients and in patients with lactation, exogenous hormone use, endocrine therapy, surgery, or radiation exposure. External validation should use locked models and should report calibration and failure cases rather than accuracy alone.

AI development should therefore address three related but distinct tasks: objective measurement of the BPE signal, identification of technical or physiologic confounders, and prediction of a clinically relevant endpoint. These tasks should not be conflated. An algorithm that detects cancer accurately in the presence of BPE is not necessarily measuring BPE, an algorithm that measures BPE reproducibly is not automatically a validated risk-prediction tool, and a risk model should not be introduced clinically until the underlying measurement has demonstrated technical stability and external reproducibility.

### 9.3. Prospective Clinical Utility and Implementation Studies

Prospective studies should determine not only whether CEM BPE is associated with cancer risk or examination performance, but also whether acting on that information improves patient outcomes. Early clinical-utility studies should use predefined BPE measurements and management thresholds and should compare BPE-informed strategies with usual care. Relevant outcomes include cancer detection, interval cancers, false-positive recalls, additional imaging, biopsy rates, contrast exposure, patient anxiety, and cost.

Implementation studies should also evaluate structured data capture, reader adherence, reporting consistency, workflow burden, patient communication, and performance across institutions, vendors, and clinically diverse populations. Particular attention should be given to equity, because a model developed in a selected screening or high-risk cohort may not perform similarly in other populations. Until a BPE-informed strategy demonstrates benefit that outweighs its potential harms, BPE should remain a descriptive and investigational variable rather than an independent trigger for clinical action.

### 9.4. Limitations of the Evidence and Review Process

The evidence on BPE at CEM is still developing and has several important limitations. The published studies included different patient populations and used CEM for a range of indications, from screening to diagnostic evaluation and treatment assessment. Acquisition techniques also varied between centers and vendors, including contrast administration, image timing, projection order, and postprocessing. In addition, BPE was not assessed consistently: some studies relied on visual categories, whereas others used quantitative methods, and reader experience and definitions differed across studies.

Most of the available evidence comes from retrospective, single-center studies, and only a small number have examined how BPE changes over time. Associations with clinical outcomes may also be influenced by age, breast density, menstrual or menopausal status, hormone use, endocrine therapy, systemic treatment, and technical factors. Publication and selection bias are possible, particularly if studies with positive findings were more likely to be published. MRI studies were used selectively to provide biologic and methodological context, but MRI findings were not treated as direct evidence for CEM. Because the studies differed substantially in their populations, protocols, BPE measurements, and outcomes, a meaningful meta-analysis could not be performed.

## 10. Conclusions

BPE on CEM is best understood as a functional imaging feature shaped by biology, tissue substrate, acquisition technique, and reader interpretation. Early studies suggest that higher CEM BPE may be associated with breast cancer risk, including density-independent associations and longitudinal dose–response findings in high-risk women. The evidence, however, remains heterogeneous, and BPE is dynamic, technically modulated, and not yet validated for independent clinical decision-making. In current practice, CEM BPE should be reported consistently, interpreted in context, and recognized as both a potential masking factor and a possible future biomarker. Multicenter work with standardized quantitative assessment and longitudinal risk validation is needed before BPE can guide management.

The practical message is therefore one of measured integration. Radiologists should not ignore CEM BPE, because it can affect lesion conspicuity, diagnostic confidence, and possibly future risk assessment. They also should not overstate it, because current evidence is not sufficient to classify an individual patient as high risk or to change management solely on the basis of BPE. The most defensible current approach is consistent reporting, careful attention to asymmetry and technical factors, and participation in standardized research that links reproducible BPE measures with clinically meaningful outcomes.

The appropriate near-term goal is not immediate BPE-directed management but disciplined preparation for validation. Practices can support this process through consistent structured reporting, documentation of relevant technical and hormonal context, reader calibration, and preservation of longitudinal CEM data. Research should progress sequentially from reproducible measurement to external clinical validation, demonstration of incremental value beyond established risk factors, and prospective evidence of clinical benefit. Only after these stages are completed should BPE be considered for use in screening or risk-management decisions.

## Figures and Tables

**Table 1:** Representative CEM BPE studies and their relevance.

Study	Population/Focus	Main Contribution for CEM BPE
Sogani et al., 2017 [11]	Women with CEM and MRI	Compared BPE across modalities; showed overlap but not identity between CEM and MRI BPE.
Sorin et al., 2020 [22]	Screening and diagnostic CEM cohort	Moderate-to-marked BPE associated with increased odds of breast cancer independent of density.
Yu et al., 2020 [24]	Diagnostic CEM cohort	No significant association between BPE and malignancy at presentation; endpoint was diagnostic, not future risk.
Karimi et al., 2021 [13]	CEM examinations	Identified clinical factors associated with BPE, supporting patient-level influences.
Wang et al., 2023 [16]	Patients undergoing CEM	Evaluated early BPE and clinical factors, reinforcing hormonal and demographic influences.
Xu et al., 2023 [23]	High-risk women followed after CEM	Reported dose–response relationship between BPE and subsequent breast cancer.
Magni et al., 2024; Moffa et al., 2025; Nicosia et al., 2025 [14,15,18]	Retrospective CEM cohorts	Expanded evidence linking BPE with density, age, menopausal status, and patient characteristics.
Wessling et al., 2024 [17]	CEM acquisition and patient factors	Highlighted procedural and patient-dependent factors influencing BPE appearance.
Nissan et al., 2025 [19]	Hormonal regulation study	Showed CEM BPE changes with menopause, lactation, HRT, and tamoxifen exposure.
Ferrara et al., 2025 [12]	CEM-MRI comparison	Reported fair-to-moderate agreement and CEM-specific effects including compression and density.
Nissan et al., 2025 [19]	Longitudinal CEM cohort	Showed greater BPE variability than mammographic density across serial examinations.
Bechyna et al., 2025 [25]	Diagnostic performance study	Evaluated how BPE affects CEM interpretation for cancer diagnosis.

**Table 2:** Practical interpretation framework for CEM BPE.

Scenario	Suggested Interpretation	Practical Implication
Minimal or mild symmetric BPE	Usually a background feature with limited masking effect.	Report according to CEM lexicon; no management change based solely on BPE.
Moderate or marked symmetric BPE	May reduce conspicuity and may reflect hormonally active or dense parenchyma.	Interpret lesions carefully; consider clinical context and comparison exams.
Asymmetric BPE without discrete lesion	May be physiologic, technical, treatment-related, or rarely lesion-associated.	Check acquisition factors, low-energy images, ultrasound correlation, and prior studies.
High BPE in premenopausal, lactating, or hormone-therapy context	Likely influenced by hormonal state.	Avoid overstating risk; document context when clinically available.
Persistent high BPE across exams	Potentially more relevant than a single high-BPE exam.	Candidate for future risk modeling; not yet an independent clinical action trigger.
Low BPE after endocrine therapy or menopause	May reflect reduced hormonal stimulation.	Potentially useful in research on therapy response or prevention biomarkers.

**Table 3:** Proposed roadmap for validating CEM background parenchymal enhancement as a clinical biomarker.

Stage	Validation Objective	Key Research Requirements
**1. Measurement reliability**	Establish that CEM BPE can be measured consistently across readers, repeated examinations, imaging systems, acquisition protocols, and institutions.	Standardized BPE definitions and scoring methods; interreader and intrareader agreement studies; repeatability studies; and multicenter technical validation.
**2. Clinical and prognostic validation**	Determine whether CEM BPE is independently associated with incident breast cancer after accounting for breast density, age, hormonal factors, family history, and other established risk factors.	Large longitudinal cohorts with adequate follow-up, standardized BPE assessment, clearly defined incident cancer outcomes, and multivariable adjustment for relevant confounders.
**3. Incremental predictive value**	Determine whether incorporating CEM BPE meaningfully improves established breast cancer risk-prediction models.	Comparison of model performance with and without BPE using discrimination, calibration, reclassification, and decision-curve analyses, followed by external validation in independent populations.
**4. Clinical utility and implementation**	Determine whether the use of CEM BPE information improves patient outcomes or clinical decision-making without producing excessive imaging, biopsies, anxiety, inequity, or cost.	Prospective clinical studies, impact analyses, cost-effectiveness evaluations, implementation studies, and assessment of patient-centered outcomes and potential harms.

**Legend:** The roadmap represents a progressive but potentially iterative validation pathway. Reliable and standardized measurement is required before prognostic associations can be interpreted confidently. Demonstration of an independent association with future breast cancer should then be followed by evidence that CEM BPE adds meaningful predictive information beyond established risk factors and existing risk models. Clinical implementation should be considered only after prospective studies demonstrate that.

## Data Availability

No new data were created or analyzed in this study. Data sharing is not applicable to this article.

## Supplementary Materials

The following supporting information can be downloaded at: https://www.mdpi.com/article/10.3390/cancers18152378/s1, Figure S1: Study-selection process. The supplied PubMed/export reported 2656 records; Table S1: Full database-specific search strategies.
