# Supplementary material for: Background Parenchymal Enhancement on Contrast-Enhanced Mammography: Determinants, Technical Considerations, and Emerging Role as a Breast Cancer Risk Biomarker
Source: Cancers (Basel). 2026 Jul 23;18(15):2378. doi: 10.3390/cancers18152378 (PMC13465418; doi:10.3390/cancers18152378)
Supplement: Supplementary file 1 [file cancers-18-02378-s001.zip › cancers-4372160-supplementary.pdf]

cancers

MDPI

Supplementary Material

Background Parenchymal Enhancement on Contrast-Enhanced Mammography

Table S1. Full database-specific search strategies.

Database / platform	Full search strategy	Limits / date
PubMed/MEDLINE (PubMed)	("contrast-enhanced mammography"[Title/Abstract] OR "contrast enhanced mammography"[Title/Abstract] OR "contrast-enhanced spectral mammography"[Title/Abstract] OR "contrast enhanced spectral mammography"[Title/Abstract] OR "contrast-enhanced digital mammography"[Title/Abstract] OR "contrast enhanced digital mammography"[Title/Abstract] OR CEM[Title/Abstract] OR CESM[Title/Abstract] OR CEDM[Title/Abstract]) AND ("background parenchymal enhancement"[Title/Abstract] OR "parenchymal enhancement"[Title/Abstract] OR BPE[Title/Abstract])	Database inception to April 2026; humans; no language restriction

Methodological note. Only the PubMed/MEDLINE results export was supplied and directly assessed.

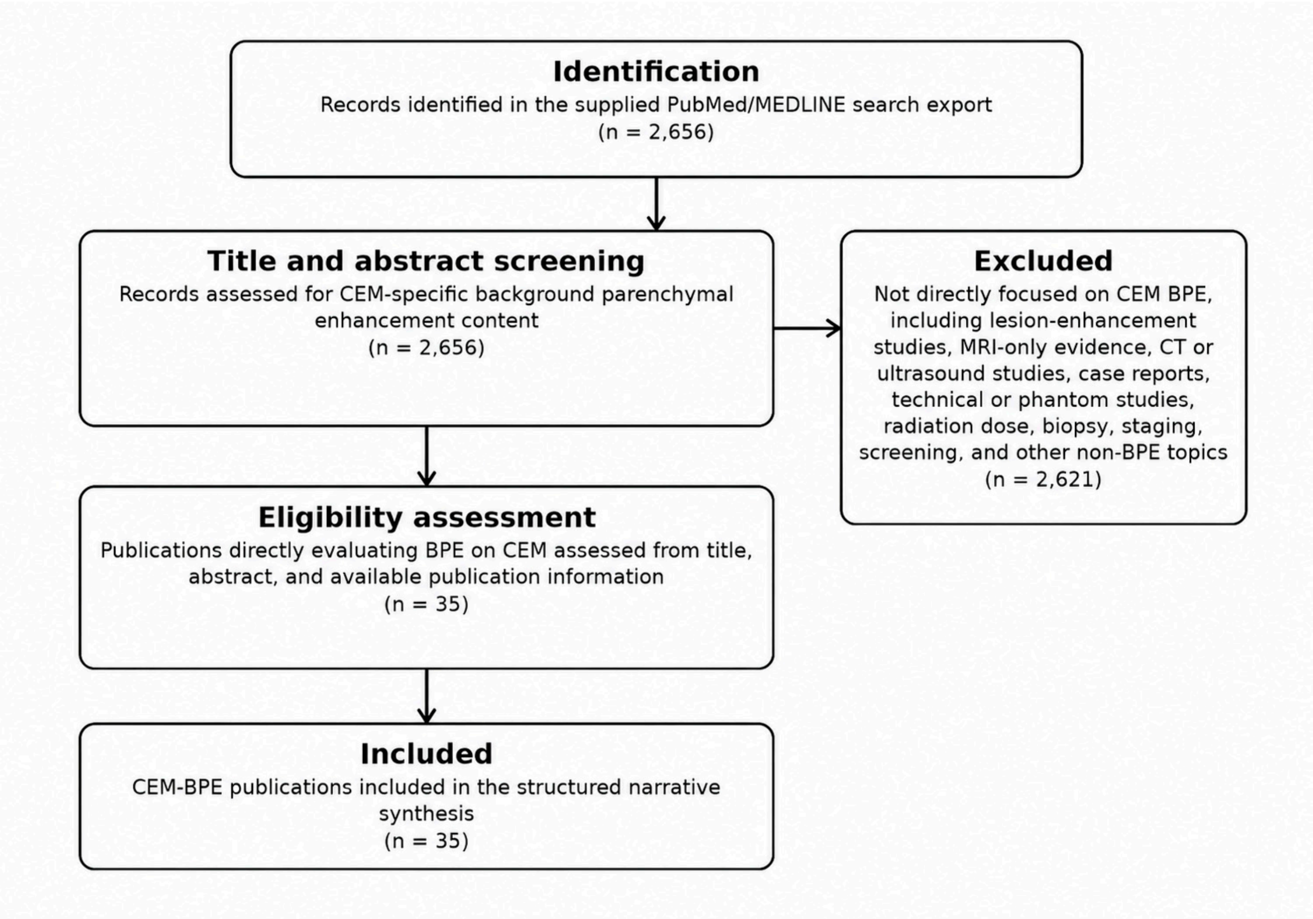

Figure S1. Study-selection process. The supplied PubMed/ export reported 2,656 records. Titles, abstracts, and available publication information were assessed for direct evaluation of background parenchymal enhancement on CEM. 35 publications were selected for the structured narrative syn-

 https://doi.org/10.3390/cancers18152378

thesis. Because the source was a broad PubMed results export rather than a deduplicated multi-database library, this figure documents the selection performed from that export and is not presented as a formal PRISMA systematic-review flow diagram.

Disclaimer/Publisher's Note: The statements, opinions and data contained in all publications are solely those of the individual author(s) and contributor(s) and not of MDPI and/or the editor(s). MDPI and/or the editor(s) disclaim responsibility for any injury to people or property resulting from any ideas, methods, instructions or products referred to in the content.

https://doi.org/10.3390/cancers18152378
